# Factors associated with individual-level changes in BMI as a result of COVID-19 in the general- and migrant-origin populations in Finland

**DOI:** 10.1177/17579759241307946

**Published:** 2025-01-20

**Authors:** T. Prinkey, A. Lundqvist, R. García Velázquez, E. Lilja, N. Skogberg

**Affiliations:** Finnish Institute for Health and Welfare, Helsinki, Finland

**Keywords:** migrant, BMI, COVID-19, snacking, physical activity, smoking, alcohol, obesity/overweight

## Abstract

**Aims:**

There is limited information on changes in body mass index (BMI) due to the COVID-19 pandemic among persons of migrant origin. The aim of the present study was to examine factors associated with changes in BMI among the general- and migrant-origin populations in Finland.

**Methods:**

Longitudinal data to explore individual-level changes in self-reported BMI among migrant-origin persons (*N* = 3313) were obtained from the FinMonik Survey conducted in 2018 and the MigCOVID Survey conducted 2020–2021. Data for the general population reference group were obtained from the FinHealth 2017 Study conducted 2017–2018 and its follow-up conducted in 2020 (*N* = 2982). Logistic regression was applied to examine whether age, sex, education, economic activity, length of residence in Finland, language skills, smoking, alcohol usage, sleep, physical activity, snacking, and fruit and vegetable consumption were associated with an increase or decrease in BMI. A change in BMI was defined as a 5% or greater increase or decrease.

**Results:**

Twenty-seven per cent of the migrant-origin population experienced an increase in BMI, while 14% had a decrease in BMI. These results corresponded to figures observed among the general population in Finland (27% and 14%). Persons of migrant origin who were other than students or employed faced greater odds of an increase in BMI of at least 5% (OR = 1.71). In the general population, an increase in BMI of at least 5% had greater odds of occurring among women (OR = 1.61), those who were other than students or employed (OR = 1.68), those who increased their alcohol intake (OR = 1.64), those who increased their snacking (OR = 1.40) and decreased their fruit and vegetable intake (OR = 1.85).

**Conclusions:**

Most examined factors applied differently to general- and migrant-origin populations and by migrant-origin group. These differences must be considered when planning future public health promotion efforts, particularly those during crisis situations.

## Introduction

Obesity has emerged as a significant public health challenge, with its prevalence steadily increasing worldwide (1). Research suggests that many experienced an increase in body weight because of COVID-19 pandemic-related restrictions (2–4). Factors associated with increases include older age (5–7), being female (5,8–10), decreased physical activity (PA) (5,11), increased snacking between meals (5,11), greater consumption of sweets (7,9–11), decreased fruit and vegetable consumption (6), increased alcohol consumption (5,7,10,12), having a higher starting weight (8,9,13), and occupational changes such as working from home (9,14) or suspending one’s usual job (9).

A total of 23% of adults in the European Region are living with obesity (i.e. body mass index (BMI) 30 kg/m^2^ or more) and it is more prevalent among women (24%) than men (22%) (15). In Finland, the prevalence of obesity is 30% among men and 27% among women (16).

The migrant-origin population in Finland has grown rapidly over the past several decades, currently constituting over 9% of the population, with the largest migrant groups being those from Russia/Former Soviet Union, Estonia, Sweden, Ukraine, Iraq and China (17). The prevalence of obesity in the migrant-origin population in Finland has been found to be 17% among men and 18% among women, which is somewhat lower than among the general population in Finland (18). However, Kurdish (i.e. born in Iran or Iraq) and Somali-origin migrant women have been found to have a higher prevalence of obesity (55% and 65%, respectively) than the general Finnish population (19). To the best of our knowledge, no studies examining changes in BMI were conducted among migrant-origin persons during the COVID-19 pandemic. This gap in information makes it challenging to identify shifting health risks in migrants, and difficult to ensure that migrants are not left behind in post-pandemic recovery efforts.

Research prior to COVID-19 among migrant-origin populations in Europe investigating increases in BMI found associations with the geographical origin of parents (20) and educational level (21). Ethnicity and age were found to be associated with weight gain among female migrants (22), while length of residence was found to be positively associated with obesity in North African migrant women (23). A lower BMI among persons of migrant origin has also been associated with higher levels of acculturation (24). Lifestyle factors associated with weight changes are less understood among migrant-origin populations. To effectively target health promotion efforts for various migrant-origin groups, it is crucial to understand the specific factors affecting BMI changes in these groups and how they differ from the general population.

The aim of this research was to fill the gap in knowledge on BMI change among migrant-origin populations in Finland. Our objectives included examining BMI changes among persons of migrant origin in Finland during COVID-19 and to investigate which factors were associated with changes in BMI. BMI is a multifactorial health outcome influenced by individual behaviours and broader socioeconomic factors. Based on the social determinants of health framework, this study included predictors such as age, sex, education, economic activity, length of residence, language skills and lifestyle factors, which have been shown to be associated with BMI in previous research.

## Methods

The current study drew upon longitudinal data collected in 2017–2019 (baseline) and 2020–2021 (follow-up), allowing for the examination of individual-level changes that occurred during the COVID-19 pandemic. The FinMonik Survey (THL/271/6.02.01/2018) and the MigCOVID Survey (THL/4061/6.02.01/2020) received ethical permission from the Ethical Committee of THL (Finnish Institute for Health and Welfare). The FinHealth 2017 follow-up study (HUS/2391/2020) received ethical permission from the Ethics Committee II of the Helsinki and Uusimaa Hospital region.

For the migrant-origin population, baseline data were collected during the Survey on Well-Being among Foreign Born Population (FinMonik) conducted between 2018 and 2019. The baseline stratified sample (*N* = 13,650) was drawn from the Finnish Population Register. Selection criteria were being aged between 18 and 64 years, residing in Finland for at least 1 year, and that both the participant and their parent(s) were born outside Finland (25). Persons who took part in the baseline FinMonik Survey and agreed to further contact later on, and who were still living in Finland as of September 2020 (*n* = 5269), were invited to take part in the follow-up Impact of the Coronavirus on the Wellbeing of the Foreign Born Population (MigCOVID) Survey, conducted during the second wave of the COVID-19 pandemic in Finland from October 2020 to February 2021 (26). Out of those invited, 3668 persons took part in the follow-up survey (participation rate 60%). The current study is comprised of a subsample of participants aged 21–66 years to match the age range of the general population reference group. Following the exclusion of item non-responses, the final study population in this paper constituted 3313 participants.

Participants received invitation letters, available in 18 languages, by mail with information on their personal login information to the electronic questionnaire. Paper-based questionnaires and telephone interviews supplemented the data collection. Language of contact was determined by the language (Finnish or Swedish) each respondent marked in the Finish Population Register and the language they had chosen to respond in during the baseline survey. Participants were able to fill out the electronic questionnaire in any of the 18 available languages. Telephone interviews were conducted in 12 languages by trained fieldwork personnel.

The reference group for the present study constituted persons who took part in both the FinHealth 2017 Study (conducted in 2017) (27) and its follow-up study conducted between late 2020 and early 2021 (*n* = 3490; participation rate 51%) and who represented the overall Finnish population (later referred to as the general population). The FinHealth 2017 Study included persons aged 18 years and older, and this random sample was drawn from the population register in 2017. A subsample of the reference group (*n* = 2982) was used in this study of adults between 21 and 66 years of age living in Finland, to match the age range of the MigCOVID Survey. The FinHealth 2017 Study does not have sufficient information to reliably distinguish persons of migrant origin as they were distinguished for the FinMonik/MigCOVID surveys. Altogether, 4.2% of the sample had a language other than the official languages in Finland, but this alone is not a reliable indicator of migrant origin.

### Variable definitions

In all the surveys, information on date of birth, age, sex and length of residence in Finland was obtained from the Finnish Population Register. Participants from the MigCOVID Survey were grouped into five regions of origin based on their country of origin: Russia or the Former Soviet Union (FSU); Estonia; the Rest of Europe, North America and Oceania; the Middle East and Africa; Asia and Latin America.

Self-reported data included information on education (less than secondary education, secondary education or greater), economic activity (full-time or part-time can work remotely, full-time or part-time cannot work remotely, student, ‘other’) and Finnish/Swedish language skills (beginner language skills or less, intermediate skills or greater). A comprehensive economic activity variable was created by combining economic activity with the ability to work remotely. The resulting economic activity groups were ‘full-time or part-time can work remotely’, ‘full-time or part-time cannot work remotely’, ‘student’, and ‘other’. The ‘other’ category applied to those who were retired on an old age pension, received disability, were on part-time retirement, were unemployed/laid off, were on family leave, or were a stay-at-home parent. The ‘other’ category will be referred to in the subsequent text as ‘other than students or employed’.

Self-perceived changes in smoking, alcohol, PA, fruit and vegetable consumption, and snacking were assessed by asking how the pandemic or its restrictive measures affected respondents’ everyday life. Participants answered by choosing ‘no effect’, ‘yes, decreased’, ‘yes, increased’ or ‘does not apply’ for each lifestyle factor. Answers ‘does not apply’ and ‘no effect’ were coded together.

BMI was calculated using self-reported height and weight in the following equation:

$$BMI=\;weight\;(kg)\;/\;{\left[height\;(m)\right]}^{2}$$

The present study followed the example of a 2017 study by Svärd *et al.* (28) that defined a significant change in BMI as ⩾ 5% change (i.e. increase or decrease) between two data collection points.

### Statistical analyses

Logistic regressions were used to investigate associations between lifestyle and sociodemographic factors and changes of 5% or greater in BMI. Two models were created: the first model adjusted for age and sex, while the second model adjusted for age, sex, education, economic activity, length of residence, language skills, smoking, alcohol use, sleep, PA, snacking, and fruit and vegetable consumption. Odds ratios (ORs) and their corresponding 95% confidence intervals (CIs) were calculated. We first examined whether lifestyle and sociodemographic factors were associated with an increase in BMI, and then whether the same or different factors were associated with a decrease in BMI.

Sample size calculations were based on an assumed prevalence of 15% and 25% of decreasing and increasing weight changes, respectively, a total of 16 beta parameters per regression model, and an expected value of Nagelkerke’s *R*^2^ of 0.10. The final (most conservative) sample requirement is provided. Separate sample size calculations were made for BMI increases and decreases. For changes indicating a decrease, the required sample size was 2446, while it was 2037 for increases. Sample sizes for both datasets in our study exceeded requirements: the migrant-origin sample size was 3313 and the general population was 2982.

In the regression models, *p*-values were calculated using the Wald test statistic. Model-adjusted proportions were calculated with predictive margins. Non-response bias was reduced by using inverse probability weights, which also took varying sampling probabilities into account and made estimates representative of the population (29).

All statistical analyses were performed using the ‘survey’ package in the R statistics software (30). Sample size calculations were performed with the ‘pmsampsize’ package (31). The significance level in all our hypothesis testing was set at α = 0.05.

## Results

The pre-pandemic obesity prevalence for the general- and migrant-origin populations was 18.1% and 15.3%, respectively, while during the pandemic the prevalence was 20.3% and 17.4%, respectively. Descriptive characteristics of the study participants are presented in Table 1. Russia/FSU had the lowest proportion of men while the highest proportion of men belonged to migrants from the Middle East and Africa. Russia/FSU and Estonia had the highest proportion of participants belonging to the oldest age group, while the Middle East and Africa as well as Asia and Latin America had the lowest.

**Table 1.** Descriptive characteristics of the study participants.

Variable	General population	Migrant-origin total	Russia or FSU	Estonia	Rest of Europe	Middle East and Africa	Asia. . .^1^
	*N* = 2982	*N* = 3313	*N* = 1085	*N* = 295	*N* = 680	*N* = 542	*N* = 711
	*n* (%)	*n* (%)	*n* (%)	*n* (%)	*n* (%)	*n* (%)	*n* (%)
Men	1354 (45.4)	1472 (44.4)	371 (34.2)	107 (36.3)	381 (56)	362 (66.8)	251 (35.3)
Age							
21–34 years	372 (12.5)	962 (29)	254 (23.4)	56 (19)	172 (25.3)	215 (39.7)	265 (37.3)
35–49 years	978 (32.8)	1398 (42.2)	398 (36.7)	117 (39.7)	331 (48.7)	230 (42.4)	322 (45.3)
50–66 years	1632 (54.7)	953 (28.8)	433 (39.9)	122 (41.4)	177 (26)	97 (17.9)	124 (17.4)
Less than secondary education	217 (7.3)	312 (9.5)	53 (4.9)	32 (11.1)	50 (7.4)	93 (17.6)	84 (12)
Economic activity							
Full-time or part-time can work remotely	873 (29.8)	655 (21)	166 (16.1)	32 (11.6)	232 (36.1)	77 (15.4)	148 (22.3)
Full-time or part-time cannot work remotely	1209 (41.3)	1445 (46.4)	511 (49.6)	173 (62.7)	251 (39)	188 (37.5)	322 (48.4)
Student	88 (3)	315 (10.1)	77 (7.5)	7 (2.5)	37 (5.8)	114 (22.8)	80 (12)
Other*	759 (25.9)	701 (22.5)	277 (26.9)	64 (23.2)	123 (19.1)	122 (24.4)	115 (17.3)
Length of residence in Finland							
3–6.99 years	N/A	892 (26.9)	192 (17.7)	45 (15.3)	158 (23.2)	229 (42.3)	268 (37.7)
7–11.99 years	N/A	940 (28.4)	272 (25.1)	119 (40.3)	196 (28.8)	131 (24.2)	222 (31.2)
12+ years	N/A	1481 (44.7)	621 (57.2)	131 (44.4)	326 (47.9)	182 (33.6)	221 (31.1)
Beginner language skills or less	N/A	989 (30.1)	235 (21.8)	34 (11.7)	225 (33.3)	169 (31.6)	326 (46.2)
Smoking increased	61 (2.1)	116 (3.5)	34 (3.1)	9 (3.1)	25 (3.7)	32 (6)	16 (2.3)
Alcohol intake increased	163 (5.5)	132 (4)	41 (3.8)	7 (2.4)	40 (5.9)	21 (3.9)	23 (3.3)
Sleeping difficulties increased	276 (9.4)	550 (16.7)	151 (14)	40 (13.7)	121 (17.9)	110 (20.7)	128 (18.2)
Physical activity decreased	963 (32.8)	1233 (37.5)	326 (30.2)	87 (29.7)	248 (36.6)	260 (48.9)	312 (44.4)
Snacking increased	608 (20.6)	672 (20.5)	196 (18.2)	43 (14.6)	172 (25.4)	109 (20.7)	152 (21.5)
Fruit and vegetable intake decreased	151 (5.1)	215 (6.6)	63 (5.8)	15 (5.1)	36 (5.3)	44 (8.3)	57 (8.1)

1 Asia group includes Asia and Latin America.

* Other: those who are retired on an old age pension, receive disability, are on part-time retirement, are unemployed/laid off, are on family leave, or are a stay-at-home parent.

Decreased PA was highest among migrants from the Middle East and Africa and lowest among migrants from Russia/FSU and Estonia. Increased snacking was highest among migrants from the Rest of Europe, North America and Oceania and lowest among migrants from Estonia.

### Distribution of BMI and 5% change in BMI adjusted by age and sex

Information on the distribution of BMI and 5% change in BMI is presented in Table 2. Changes in BMI in this study ranged from 37.9% to 42.9% among migrant groups. The 5% increases in BMI ranged from 24.0% to 30.1%, and 5% decreases ranged from 11.3% to 15.2%. Migrants from Russia/FSU had a significantly lower mean BMI in 2020 when compared to the general population. Migrants from the Middle East and Africa had a significantly higher mean BMI in both 2018 and 2020, while migrants from Asia and Latin America had significantly lower mean BMIs in both 2018 and 2020 compared to the general population.

**Table 2.** Distribution of BMI and 5% change in BMI adjusted by age and sex.

BMI metrics	General population	Migrant-origin total	Russia or FSU	Estonia	Rest of Europe	Middle East & Africa	Asia . . .^1^
	*n* = 2982	*n* = 3313	*n* = 1085	*n* = 295	*n* = 680	*n* = 542	*n* = 711
Mean BMI 2018 kg/m^2^ (95% CI)	26.01 (25.77–26.25)	25.81 (25.49–26.13)	25.81 (25.15–26.47)	26.79 (25.72–27.86)	25.69 (25.18–26.20)	27.06 (26.37–27.75)***	23.84 (23.29–24.39)***
Mean BMI 2020 kg/m^2^ (95% CI)	26.40 (26.18–26.63)	26.21 (25.88–26.55)	25.78 (25.31–26.25)*	26.98 (25.90–28.07)	26.59 (25.70–27.47)	27.49 (26.78–28.20)***	24.41 (23.88–24.93)***
Mean change BMI kg/m^2^ (95% CI)	0.40 (0.28–0.51)	0.41 (0.14–0.67)	−0.03 (−0.55–0.50)	0.19 (−0.21–0.59)	0.89 (0.19–1.60)	0.43 (−0.29–1.14)	0.56 (0.18–0.94)
BMI 5% change % (95% CI)	41.2 (38.4–44.2)	40.8 (37.9–43.9)	37.9 (33.1–43.4)	42.9 (34.8–52.9)	41.5 (35.5–48.5)	40.4 (34.0–48.0)	42.4 (36.1–49.9)
Increase % (95% CI)	27.3 (24.7–30.1)	27.1 (24.5–29.9)	24.0 (20.1–28.6)	28.4 (21.3–37.8)	30.1 (24.4–37.2)	26.6 (21.1–33.6)	27.2 (21.3–37.8)
Decrease % (95% CI)	13.9 (11.8–16.4)	13.8 (11.9–15.9)	13.9 (10.7–18.1)	14.5 (9.1–23.1)	11.3 (8.2–15.7)	13.8 (9.9–19.2)	15.2 (11.3–20.5)

1 Asia group includes Asia and Latin America.

*P*-values were calculated for the differences between migrant group and general population.

* *p*-value < 0.05; **p-value < 0.01; ***p-value < 0.001.

#### Logistic regression analysis of factors associated with 5% or greater increases in BMI

Information on factors associated with 5% increases in BMI is presented in Table 3. In the general population, 27.3% experienced an increase in BMI whereas 27.1% of the migrant-origin population experienced an increase. In Model 2 for the migrant-origin population, an increase in BMI of at least 5% had 1.71 times greater odds of occurring among those who were other than students or employed.

**Table 3.** Logistic regression analysis of factors associated with a 5% or greater increase in BMI.

Factors	General population		Migrant origin	
	Model 1^1^	Model 2^2^	Model 1^1^	Model 2^2^
	OR (95% CI)	OR (95% CI)	OR (95% CI)	OR (95% CI)
Sex				
Men	1.00	1.00	1.00	1.00
Women	1.72 (1.35–2.19)***	1.61 (1.26–2.07)***	1.00 (0.76–1.32)	1.01 (0.75–1.36)
Age				
21–34	1.00	1.00	1.00	1.00
35–49	0.77 (0.53–1.12)	0.75 (0.54–1.03)	0.63 (0.46–0.87)**	0.69 (0.49–0.98)*
50–66	0.45 (0.31–0.66)***	0.39 (0.27–0.57)***	0.49 (0.34–0.69)***	0.55 (0.36–0.83)**
Education				
Basic education or less	1.00	1.00	1.00	1.00
Secondary or higher	0.58 (0.42–0.80)***	0.61 (0.43–0.86)**	0.50 (0.32–0.77)**	0.65 (0.41–1.03)
Economic activity				
Full-time or part-time can work remotely	1.00	1.00	1.00	1.00
Full-time or part-time cannot work remotely	1.17 (0.91–1.50)	1.23 (0.94–1.6)	1.07 (0.75–1.50)	0.97 (0.64–1.47)
Student	0.98 (0.42–2.25)	1.01 (0.43–2.37)	1.62 (1.01–2.59)*	1.14 (0.67–1.96)
Other	1.62 (1.21–2.17)***	1.68 (1.26–2.25)***	1.94 (1.31–2.88)***	**1.71 (1.18–2.48)** **
Length of residence in Finland				
3–6.99 years	1.00	1.00	1.00	1.00
7–11.99 years	N/A	N/A	0.89 (0.63–1.25)	0.85 (0.56–1.29)
12+ years	N/A	N/A	0.79 (0.57–1.08)	0.76 (0.5–1.14)
Language skills				
Intermediate or advanced	1.00	1.00	1.00	1.00
Beginner or less	N/A	N/A	1.10 (0.83–1.46)	0.81 (0.57–1.15)
Smoking				
No change/decreased/does not apply	1.00	1.00	1.00	1.00
Increase	0.80 (0.40–1.61)	0.50 (0.26–0.96)*	2.68 (1.37−5.28)**	1.3 (0.64–2.64)
Alcohol				
No change/decreased/does not apply	1.00	1.00	1.00	1.00
Increased	1.75 (1.17–2.62)**	1.64 (1.06–2.54)*	1.45 (0.79–2.65)	1.35 (0.67–2.75)
Sleep				
No change/increased/does not apply	1.00	1.00	1.00	1.00
Decreased	1.33 (0.89–1.97)	1.11 (0.74–1.67)	1.40 (0.98–1.99)	1.16 (0.75–1.79)
Physical activity				
No change/increased/does not apply	1.00	1.00	1.00	1.00
Decreased	1.04 (0.82–1.32)	1.02 (0.79–1.32)	0.86 (0.66–1.14)	0.79 (0.58–1.09)
Snacking				
No change/decreased/does not apply	1.00	1.00	1.00	1.00
Increased	1.57 (1.16−2.11)**	1.4 (1.04–1.9)*	1.26 (0.91–1.73)	1.37 (0.96–1.96)
Fruit and vegetable consumption				
No change/increased/does not apply	1.00	1.00	1.00	1.00
Decreased	1.90 (1.23–2.95)**	1.85 (1.18–2.9)**	1.16 (0.69–1.96)	0.79 (0.43–1.45)

1 Model 1 is adjusted for age and sex.

2 Model 2 is adjusted for all included variables.

* *p*-value < 0.05; **p-value < 0.01; ***p-value < 0.001

In Model 2 for the general population, an increase in BMI of at least 5% had greater odds of occurring among women (OR = 1.61), those who were other than students or employed (OR = 1.68), those who increased their alcohol intake (OR = 1.64), who increased their snacking (OR = 1.40) and decreased their fruit and vegetable intake (OR = 1.85).

#### Logistic regression analysis of factors associated with 5% or greater decreases in BMI

Information on factors associated with 5% decreases in BMI is presented in Table 4. In the general population, 13.9% experienced a decrease in BMI, whereas 13.8% of the migrant-origin population experienced a decrease. In Model 2 for the general population, a decrease in BMI of at least 5% had greater odds of occurring among students (OR = 2.14) and those who increased their smoking (OR = 2.49).

**Table 4.** Logistic regression analysis of factors associated with a 5% or greater decrease in BMI.

Factors	General population		Migrant origin	
	Model 1^1^	Model 2^2^	Model 1^1^	Model 2^2^
	OR (95% CI)	OR (95% CI)	OR (95% CI)	OR (95% CI)
Sex				
Men	1.00	1.00	1.00	1.00
Women	0.75 (0.54–1.04)	0.85 (0.62–1.16)	1.4 (0.99–1.98)	1.29 (0.9–1.85)
Age				
21–34	1.00	1.00	1.00	1.00
35–49	0.79 (0.46–1.34)	1.11 (0.7–1.77)	1.17 (0.78–1.76)	1.16 (0.72–1.87)
50–66	0.89 (0.51–1.57)	1.14 (0.68–1.89)	1 (0.63–1.59)	0.99 (0.54–1.83)
Education				
Basic education or less	1.00	1.00	1.00	1.00
Secondary or higher	0.67 (0.46–0.98)*	0.77 (0.51–1.17)	1.92 (1.16–3.17)*	1.57 (0.77–3.2)
Economic activity				
Full-time or part-time can work remotely	1.00	1.00	1.00	1.00
Full-time or part-time cannot work remotely	1.08 (0.79–1.48)	0.98 (0.69–1.38)	1.02 (0.66–1.59)	0.82 (0.51–1.33)
Student	2.09 (0.98–4.46)	2.14 (1.01–4.54)*	1.33 (0.74–2.38)	1.27 (0.66–2.45)
Other	1.29 (0.92–1.81)	1.12 (0.75–1.67)	0.92 (0.58–1.48)	1 (0.62–1.61)
Length of residence in Finland				
3–6.99 years	1.00	1.00	1.00	1.00
7–11.99 years	N/A	N/A	1.01 (0.67–1.51)	1.15 (0.68–1.95)
12+ years	N/A	N/A	1.07 (0.71–1.62)	1.01 (0.6–1.71)
Language skills				
Intermediate or advanced	1.00	1.00	1.00	1.00
Beginner or less	N/A	N/A	0.86 (0.61–1.21)	0.93 (0.63–1.38)
Smoking				
No change/decreased/does not apply	1.00	1.00	1.00	1.00
Increased	1.85 (0.97–3.52)	2.49 (1.28–4.84)**	0.67 (0.31–1.45)	0.73 (0.3–1.79)
Alcohol				
No change/decreased/does not apply	1.00	1.00	1.00	1.00
Increased	0.73 (0.41–1.31)	0.85 (0.46–1.58)	1.03 (0.49–2.19)	1.41 (0.59–3.37)
Sleep				
No change/increased/does not apply	1.00	1.00	1.00	1.00
Decreased	0.94 (0.55–1.62)	1.15 (0.66–2.01)	1.03 (0.64–1.65)	1.22 (0.73–2.03)
Physical activity				
No change/increased/does not apply	1.00	1.00	1.00	1.00
Decreased	0.69 (0.47–1.01)	0.73 (0.48–1.12)	0.80 (0.56–1.14)	0.98 (0.67–1.44)
Snacking				
No change/decreased/does not apply	1.00	1.00	1.00	1.00
Increased	0.40 (0.28–0.59)***	0.42 (0.27–0.65)***	0.75 (0.47–1.18)	0.80 (0.48–1.31)
Fruit and vegetable consumption				
No change/increased/does not apply	1.00	1.00	1.00	1.00
Decreased	0.80 (0.47–1.37)	1.01 (0.57–1.77)	0.72 (0.41–1.27)	0.89 (0.4–1.96)

1 Model 1 is adjusted for age and sex.

2 Model 2 is adjusted for all included variables.

* *p*-value < 0.05; **p-value < 0.01; ***p-value < 0.001.

## Discussion

Previous research on prevalence of change in BMI during the COVID-19 pandemic found a large range of changes in BMI across Europe (2–4). This study confirmed the high prevalence of change in BMI found across Europe. This new information is consistent with what is currently known of prevalence in BMI during the pandemic and fills a gap in information about migrant-origin groups, which is severely limited in Europe.

Our study found an increase in BMI was associated with younger age and being other than economically active or a student in both the general and migrant-origin populations. These associations may be the result of increases in sedentary behaviour (32) and decreases in PA (33) due to lifestyle disruptions. Moreover, PA in Sweden was found to have decreased the most among the youngest and oldest age groups (33).

Our results are consistent with previous research that discovered that increases in BMI during the pandemic were associated with being female (5,8–10), increased snacking between meals (5,12), a greater consumption of sweets (5,7,9,10), decreased fruit and vegetable consumption (6) and increased alcohol consumption (5,7,10,12). Although our results support these findings among the general population, these factors were not significant among persons of migrant origin. The lack of significance for increased snacking may be partly explained by the potential for type II error as the observed CIs suggested a small *p*-value. Non-significance among the remaining factors could be explained by the inability to quantify how much of a change occurred at the individual level (e.g. how much more alcohol did one drink if they reported increased alcohol consumption?).

A major strength of this research is its use of a population-representable sample conducted in multiple languages by trained multilingual personnel. To the best of our knowledge, this is the first study to examine changes in BMI during COVID-19 among migrant-origin populations and the only population-based datasets that allow for examining individual-level changes among migrant-origin populations using population-based survey data collected prior to and during the pandemic. Moreover, the extensive survey data made it possible to comprehensively consider key social determinants of health and health-related variables previously shown to be associated with obesity in individual studies.

One limitation of this study is that the non-response bias remained large, as only 26% of the original FinMonik sample was included in the analysis. Although the data used provided us with a fair understanding of how persons of migrant origin fared during the pandemic, the data lacked the context of ethnicity and race, which should be viewed as interrelated terms essential for confronting health inequities (34). Moreover, although the data size was large, it was not feasible to disaggregate the data by more specific regions of origin.

Intersectionality, or the interconnected nature of social categorisations such as race, class and gender, regarded as creating overlapping and interdependent systems of discrimination or disadvantage (35), should be examined more closely in future research as those whose data were included in this study could represent these overlapping and interdependent systems.

Previous research exploring migration-related health inequalities found these inequalities to vary according to gender, occupational status, generation as well as the health outcome studied in Europe (36). This study adds to the understanding of the health outcome BMI among persons of migrant origin and offers some insight into the factors related to health inequities.

## Conclusions

This is the only research focusing on changes to BMI among migrants due to COVID-19-related restrictions in Europe, and is the first article on the subject in Finland. This research has provided a stronger knowledge base for future public health promotion efforts by highlighting which factors were relevant to each population and how these factors differed between migrant groups. In this research we found that the factors associated with increases and decreases in BMI differed tremendously between the general population and the migrant-origin groups. This finding demonstrates the need for additional research targeting migrant-origin populations.

Despite adding significantly to research on how the COVID-19 pandemic affected persons of migrant origin, we still largely do not know the long-term effects of COVID-19 on BMI. Future studies should consider the prolonged consequences of pandemic-related restrictions on smoking, alcohol, sleep, PA, snacking, fruit and vegetable consumption and BMI.
